# Lessons Learned From Successful Papanicolaou Cytology Cervical Cancer Prevention in the Socialist Republic of Vietnam

**DOI:** 10.1002/dc.21655

**Published:** 2011-03-10

**Authors:** Eric J Suba, Stephen S Raab

**Affiliations:** 1 Department of Pathology, Kaiser Permanente Medical CenterSan Francisco, California; 2 Department of Pathology, University of Colorado Health Sciences CenterAurora, Colorado

**Keywords:** Papanicolaou cytology screening, cervical cancer prevention, developing countries, HPV vaccines, Bill & Melinda Gates Foundation

## Abstract

In 1996, we documented that the burden of cervical cancer in Vietnam was associated with troop movements during the Vietnam War. Subsequently, establishment of Papanicolaou screening in southern Vietnam was associated with reductions in cervical cancer incidence from 29.2/100,000 in 1998 to 16/100,000 in 2003. This is one of the first English-language reports of a real-world cervical cancer prevention effort associated with a decisive impact on health outcomes in a contemporary developing country. Lessons learned: if our ideological commitment is to improve health outcomes as rapidly as possible among as many people as possible, then Papanicolaou screening (with or without HPV or visual screening) must be implemented without further delay in any setting where cervical screening is appropriate but unavailable; consideration must be given to HPV vaccination after, rather than before, full coverage of target demographic groups by screening services has been achieved and/or the possibility has been excluded that HPV vaccination may be ineffective for cancer prevention. Competing ideological commitments engender imprudent yet commercially useful alternative strategies prone to decelerate global reductions in mortality by suppressing the more-rapid uptake of less-expensive open-source technology in favor of the less-rapid uptake of more-expensive proprietary technologies with uncertain real-world advantages and unfavorable real-world operational limitations. Global cervical cancer prevention efforts will become more effective if global health leaders, including the Bill & Melinda Gates Foundation, embrace an ideological commitment to improving health outcomes as rapidly as possible among as many people as possible and assimilate the policy implications of that commitment.

Before Papanicolaou cytology cervical cancer prevention services became widely available in the United States, cervical cancer was a leading cause of death among American women, with an incidence rate in 1947 of 44/100,000.^1^ Cervical cancer remains a leading cause of death in many developing countries because of a lack of population coverage by cervical screening services in these settings. American volunteers interested in cervical cancer prevention first visited Vietnam in January 1994,^2^ and were promptly presented with population-based tumor registry data documenting cervical cancer incidence rates five times higher in southern Vietnam^3^ relative to northern Vietnam.^4^ A 1996 case-control study sponsored by Stanford University documented that these regional variations in cervical cancer incidence rates were associated with prior troop movements during the Vietnam War.^5^

It is axiomatic that successful cervical cancer prevention requires adequate population coverage by mass screening, which in turn requires indigenous resources mobilized by more-progressive in-country leaders. Any role for foreign aid is correspondingly uncertain.^6^ Progress in any setting is optional, and there is genuine lack of support for cervical cancer prevention efforts within the political structures of many developing countries.^7^ In Vietnam, improvised volunteer grassroots methods were used to explore the power structure of the Vietnamese health system in efforts to identify more-progressive Vietnamese leaders and to assist their efforts to mobilize indigenous resources for cervical screening services.^2^ Vietnamese leaders decisively committed to Papanicolaou screening for cervical cancer prevention in Vietnam during the 1997 National Conference on Cancer Prevention and Control.^2^ The strategy adopted to pursue success in Vietnam was to increase consumer demand for Papanicolaou screening services while lowering their price. Vietnamese leaders mobilized consumer demand by energetically promoting the benefits of Papanicolaou screening to the Vietnamese medical community and general public, in conjunction with the establishment of population-based Papanicolaou screening demonstration projects in five strategically important districts in Ho Chi Minh City (the largest city in southern Vietnam; population ∼9 million). Concurrently, highly cost-effective fine-needle aspiration biopsy services were introduced to the Ho Chi Minh City Cancer Center,^8^ one of the largest oncology referral centers in Asia. Cervical cancer incidence rates in northern Vietnam during the 1990s were judged insufficiently high to warrant the initiation of mass screening,^9^ although, to our knowledge, there is no consensus regarding disease incidence rates below which screening should not be initiated. Volunteer American support for Vietnamese screening efforts continues to be improvised and has consisted of professional advice, much of which has subsequently been peer-reviewed and published, and multidisciplinary professional training. American participation in Vietnam's cervical screening efforts has been organized around a 501(c)(3) nonprofit corporation that has received an average of US$10,000 in annual cash donations since its establishment in 1996.^10^

De novo establishment of Papanicolaou cytology cervical cancer prevention services in Ho Chi Minh City^2^, ^9^ was associated with subsequent reductions in cervical cancer incidence from 29.2/100,000 in 1998 to 16/100,000 in 2003 (Fig. 1).^11^–^13^ Breast cancer incidence rates increased substantially during the same time interval. This is one of the first English-language reports of a real-world cervical cancer prevention effort associated with a decisive impact on health outcomes in a contemporary developing country. Publication of data linking war to disease was delayed for 8 years in an attempt to ease the process of reconciliation by presenting what most would acknowledge to be a remedy in advance of what some will perceive to be an accusation.^7^ Publication of culturally sensitive data was carefully coordinated with seminars conducted in Hanoi and Ho Chi Minh City in 2004, which explained the scientific basis for the association between war and cervical cancer and offered suggestions, based on prior observations by American social critic Peter Marin,^14^ that guilt may be an inevitable consequence of human activity, rather than a condition to be escaped or denied, and that the complexities of guilt and responsibility can only truthfully be discussed in the context of a loving struggle among individuals who retain solidarity with one another.^15^ In response to justifiable concerns voiced by influential Vietnamese health leaders regarding similarities between the harm associated with Agent Orange and the harm associated with the link between war and cervical cancer,^16^ activist civil-rights attorney David R. Richards examined medicolegal aspects of the association between the Vietnam War and cervical cancer and presented his findings at the February 2005 Annual Meeting of the Papanicolaou Society of Cytopathology. It was Mr. Richards' opinion that a June 2004 U.S. Supreme Court decision (*Sosa v. Alvarez-Machain*) appeared to foredoom civil action against American interests by Vietnamese women with cervical cancer, and that at the heart of the matter was the Alien Tort Statute of 1789 (ATS). Validation of Mr. Richards' opinion was provided in March 2005 with the dismissal of a class-action lawsuit that had referenced the ATS and charged that American corporations had committed war crimes against Vietnamese citizens by manufacturing and distributing Agent Orange.^17^

**Fig 1:**
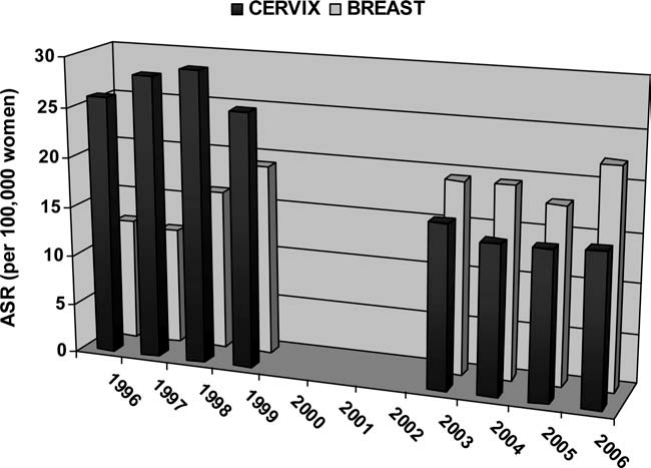
Ho Chi Minh City (population ∼9 million) population-based tumor registry data. Age-standardized incidence rates (ASR) per 100,000 women for cervical cancer and breast cancer in metropolitan Ho Chi Minh City, 1996–2006.^3^, ^11^–^13^ The first year for which population-based tumor registry data are available is 1996.^3^ Vietnamese leaders committed to Papanicolaou screening for Vietnam during the1997 National Conference on Cancer Prevention and Control.^2^ Tumor registry data from 2000, 2001, and 2002 are not yet available.^13^ The most recent year for which tumor registry data are available is 2006.^13^

No further reductions in cervical cancer incidence have been documented since 2004 in southern Vietnam (Fig. 1). Disease prevention requires social change, which in turn requires the participation of those for whom the change is intended, including high-risk demographic groups, appropriate governmental authorities, and essential medical personnel.^7^ Many forces can stall the process of major change far short of its intended goal, including turnover of key change agents and exhaustion on the part of leaders.^18^ Positive deviance (PD) is a change tool appropriate for addressing complex, intractable problems requiring behavioral and social change, and is based on the observation that, in most settings, there are individuals or groups whose uncommon behaviors and strategies enable them to find more effective solutions than peers who face similar barriers and challenges.^19^ PD identifies successful “outlier” behaviors and strategies and encourages their adoption by others. Using an exercise in PD, we identified lessons learned from Vietnam over the past 16 years as part of efforts to renew successful cervical cancer prevention in Vietnam, and to extend what success had once been achieved there to other low-resource settings.

## Methods

Exercises in PD, which have also been used to address childhood malnutrition in Vietnam,^20^ involve four basic steps described in detail elsewhere^19^: to define the problem and desired outcomes; to identify PD and referent groups; to discover uncommon but successful behaviors and strategies through a PD inquiry; and to design activities that allow others to practice successful behaviors or strategies. Preliminary findings from our PD inquiry were presented at the Global Health Council 36th Annual Conference (Washington DC, May 2009), the 1st PathTech Congress (Durban, South Africa, September 2009), the 27th Congress of the Latin American Society of Pathology (Antigua, Guatemala, November 2009), the American Public Health Association 137th Annual Meeting (Philadelphia PA, November 2009), the 5th International Conference of the Asian Pacific Organization for Cancer Prevention (Istanbul, Turkey, April 2010), and the Papanicolaou Society Anatomic Pathology Update Seminar Series (Ho Chi Minh City, Hue, and Hanoi, Vietnam, April 2010). Feedback from conference participants was incorporated into the final results of our PD exercise, which were presented at the 2010 National Conference on Cancer Prevention and Control (Hanoi, Vietnam, October 2010).^21^

## Results

### Step A: Definition of the Problem and Desired Outcomes

Regional burdens of cervical cancer are usually defined by cervical cancer incidence rates measured by population-based tumor registries, which became fully operational in Hanoi in 1988^4^ and in Ho Chi Minh City in 1995.^3^ Although reductions in cervical cancer incidence over time are generally accepted as the desired outcome of cervical screening efforts, outcomes measurements are rife with practical shortcomings in developing countries: cervical cancer incidence rates have been observed to spontaneously decline in the absence of any screening activity; improved registration methods can mask genuine declines in cervical cancer rates attributable to screening programs; and measurements of cervical cancer rates over time, even when accurate, are of little use in identifying specific programmatic improvement opportunities.^22^ Process measurements, including screening test volumes, screening test coverage rates, and followup treatment information, are therefore of critical importance in developing countries.^22^ A survey conducted in southern Vietnam documented that the three largest public-sector laboratories in Ho Chi Minh City processed more than 220,000 Papanicolaou tests in 2008.^23^ This figure does not include test volume from smaller public-sector laboratories and private-sector laboratories, and therefore significantly underestimates total screening test volume in southern Vietnam. Although organized screening coverage rates are probably insufficient to fully account for observed declines in cervical cancer incidence in southern Vietnam,^7^ opportunistic screening coverage rates in Vietnam have not yet been measured. The United States did not begin estimating opportunistic screening coverage rates until 1975.^24^

### Step B: Identification of PD and Referent Groups

For the purposes of this study, Papanicolaou cytology cervical cancer prevention in Vietnam is considered a successful outlier, and participants in Vietnamese Papanicolaou screening efforts are considered the PD group. The Bill & Melinda Gates Foundation (BMGF), which is a major contributor to global health with enormous financial power and policy leverage,^25^ and the Alliance for Cervical Cancer Prevention (“the Alliance”), established in 1999 with a gift of US$50 million from the BMGF,^7^ are considered the referent group.

### Step C: Uncommon but Successful Behaviors and Strategies Discovered Through PD Inquiry

A PD inquiry is a variant of a root cause analysis that determines through a persistent series of “why” questions what happened, why it happened, and what to do to ensure that it happens again. PD inquiry discovered that the critical behavior characterizing the PD group was an acquired ideological commitment to improving health outcomes as rapidly as possible among as many people as possible. The critical strategy characterizing the PD group was implementing Papanicolaou screening without delay in settings plagued by high rates of cervical cancer. The linkages between the behavior and strategy of the PD group are summarized as five lessons learned.

1. *Opportunity costs, borne by those less privileged, are associated with prioritizing research on novel interventions in any setting where established interventions are feasible but unavailable.*^7^, ^26^ Approximately 80% of all premature deaths in developing countries are preventable through established interventions, and what is unclear is how to make established interventions more widely available to the people who most need them.^27^, ^28^ The debate over the most appropriate strategy to assure cervical cancer prevention for all the world's women^29^, ^30^ may be considered part of a larger debate, previously articulated by President Jimmy Carter, over whether the BMGF is enamored with the promise of new technologies at the expense of delivering available preventives today.^30^ Others have criticized as potentially harmful the ideological commitment of the BMGF to novel technologies as the best route for improving health outcomes in developing countries, because this ideological commitment explicitly ignores the sociopolitical and power structure changes necessary to redistribute resources within and between societies, and is based on the doubtful premise that the problems of global health are rooted in a shortage of scientific knowledge.^27^

2. *Root cause analysis shows that critical real-world obstacles to successful cervical cancer prevention involve people far more than technology* (Table I).^7^ Because the true causes of problems are often hidden behind more obvious symptoms, root cause analyses must be used to inform the best routes for improving health outcomes among populations for the same reasons that compulsive diagnostic workups must be used to inform the best routes for improving health outcomes among individuals.^30^ Root cause analysis indicates that, locally and globally, critical real-world obstacles to successful cervical cancer prevention involve people far more than technology and present themselves as puzzles with the structure of the “prisoner's dilemma” in game theory (Table I). Cervical cancer prevention programs fail when participants uniformly pursue rational self-interest, but succeed when some participants act in manners partly contrary to rational self-interest.^7^ For example, in 1996, local fee schedules in Vietnam included costs for Papanicolaou tests which, from the perspective of pathologists with prior experience in laboratory cost analysis, lacked face validity yet presented critical obstacles against the achievement of mass screening.^30^ All workers have incentives to increase personal incomes, and the decision by more-progressive Vietnamese pathologists and health leaders to utilize time-motion studies, rather than local fee schedules, to determine costs for Papanicolaou tests in Vietnam constitutes one example of a negotiated solution to the prisoner's dilemma.^30^ Quality management, the goal of which is to confirm that women in targeted demographic groups are screened and receive appropriate followup care,^22^ is a clinical discipline that also provides solutions for the prisoner's dilemma. A lack of management skills, rather than a lack of appropriate technology, appears to be the single most important barrier to improving health throughout the world.^31^ Governmental regulation also provides solutions for the prisoner's dilemma and will probably see an expanded role in Vietnam.

**Table I:** The Prisoner's Dilemma: System Map of Real-World Obstacles to Successful Cervical Cancer Prevention^7^

*Program group (quality goal)*	*Clients*	*Competing incentives*	*Quality measures*	*Obstacles to success*
High-risk women (100% coverage)	—	Higher prices for screening visits reduce screening coverage rates	Population registers linked to cytology, histology, and/or HPV lab records	Higher net reimbursement for any group increases screening visit prices and reduces screening coverage rates
Screening test collectors (100% coverage of high-risk demographic groups)	Public health departments and private sector patients	Collecting Pap smears in private rather than public sector increases net reimbursement	Laboratory data linked to population registers	Reimbursement often inversely linked to screening coverage rates
Laboratory personnel (diagnostic accuracy)	Public health departments and private sector providers	Decreasing time spent analyzing each Pap smear or HPV test increases net reimbursement	Laboratory data analysis	Reimbursement often inversely linked to accuracy
Dysplasia treatment personnel (examine 100% of women with HGSIL or carcinoma on Pap)	Public health departments and private sector patients	Treating patients in private rather than public sector increases net reimbursement	Laboratory data analysis	Reimbursement often inversely linked to treatment of women in high-risk groups
Public health departments (goals defined by political leaders)	Political leaders	Competing sources of mortality (e.g. HIV disease, malaria, tuberculosis, avian influenza)	Budgetary allocation from government	Goals of political leaders often not linked to screening coverage rates
Academic investigators and nongovernmental organization (NGOs) (goals defined by ideological commitments of grant donors, corporate sponsors, and academic journals)	Grant donors and corporate sponsors	Fundraising and publications are required for academic career advancement and financial sustainability of NGOs	Grants and publications	Grant donor goals, corporate sponsor goals, and academic journal publication acceptance criteria often not linked to screening coverage rates
Monolayer cytology and HPV test manufacturers (goals defined by equity stakeholders)	Equity stakeholders	Higher product price increases corporate profit but lowers programmatic participation	Stock price	Equity stakeholder reward often not linked to screening coverage rates
HPV vaccine manufacturers (goals defined by equity stakeholders)	Equity stakeholders	Vaccines will not eliminate screening requirements and may compete with screening for public health budgets	Stock price	Equity stakeholder reward often not linked to screening coverage rates; HPV vaccine introduction may reduce screening coverage rates

3. *It is unlikely that more recent technological innovations will substantially improve on the performance of Papanicolaou cytology for cervical cancer prevention.* Process measurements, in conjunction with root cause analyses, will be required to determine the true causes of the deceleration in reductions of cervical cancer incidence in Vietnam. Although the true causes for the deceleration are not yet known, noncytologic preventive technologies are unlikely to be among the remedies. The U.S. Preventive Services Task Force (USPSTF) has determined that Papanicolaou screening reduces cervical cancer rates by 60%–90% within 3 years of its introduction to populations naïve to screening, and that these reductions of incidence and mortality are “consistent and dramatic across populations.”^32^ It is correspondingly unlikely that more recent technological innovations, including human papillomavirus (HPV) screening and HPV vaccines, will substantially improve on the performance of Papanicolaou screening for cervical cancer prevention. The saga of liquid-based cytology, which has largely replaced Papanicolaou cytology in the United States, has been a public health setback that should be added to the list of cautionary tales in women's health and make us more skeptical about claims of superiority for new tests and treatments, which are often based on flawed scientific methodology and amount to little more than advertising.^33^

The USPSTF has determined that the evidence is currently insufficient to recommend for or against the routine use of HPV tests as primary screening tools.^34^ Most studies of HPV testing reported from developing countries compare the performance of Papanicolaou tests analyzed in developing-country laboratories to Hybrid Capture 2® (HC2®) tests shipped to American or European reference laboratories for analysis,^35^–^38^ and may therefore be considered biased in favor of HPV testing.^26^ The performance characteristics of any screening test are to some extent operator-dependent and locality-specific, and interim analysis of data sets from large European randomized trials currently in progress show that detection rates of cervical intraepithelial neoplasia grade 3 (CIN3) by HPV testing were significantly increased relative to cytology in two trials, but not in three others.^39^ Because 25%–50% of CIN3 lesions progress to carcinoma,^40^, ^41^ epidemiologically perfect cervical screening tests (i.e., which detect 100% of lesions destined to progress and 0% of lesions destined not to progress to carcinoma) will demonstrate analytic sensitivities of 25%–50% when, as many investigators deem appropriate,^42^ CIN3 is used as the surrogate end point for cervical cancer risk. Significant differences in cervical screening test sensitivity, measured using the best available methodology, may therefore produce no real-world differences in health outcomes.

A controversial Alliance study of cervical screening in India incorporates an ongoing no-screening arm with disturbing similarities to the discredited Tuskegee Syphilis^7^ and New Zealand National Women's Hospital studies.^43^ The Alliance India study documented that Papanicolaou cytology tests analyzed by Indian cytologists with only 3 months' training performed with equal sensitivity and higher specificity than HC2® analyzed in India.^44^ However, women in the HC2® screening study arm experienced significantly less cervical cancer-related mortality than women in the Papanicolaou screening study arm. Because it is not possible for an effect to result from a cause which does not exist, it has been argued that differences in cervical cancer-related mortality among women in the different screened groups must be attributable to undocumented differences in followup care and/or screening test group biases, rather than to nonexistent differences in screening test sensitivity.^43^, ^45^–^47^

More importantly, because the unit price of HC2® (US$20–US$30/test) precludes its widespread use in low-resource settings, research on HC2® in developing countries is of uncertain relevance to people who live in developing countries. Hologic's Cervista®, Roche's Amplicor®, and Abbott's RealTime High Risk HPV tests are similarly unaffordable. The CareHPV™ test performed with equal sensitivity but far lower specificity than cytology when both tests were analyzed in China.^48^ Moreover, the International Agency for Research on Cancer warns that “increased competition resulting in diminishing market share and reductions in the cost of testing might lead HPV test manufacturers to relax their standards of quality. Such a scenario could prove disastrous in many respects, since there are theoretically many more variables that can affect the performance of HPV testing than there are for cytology-based screening.”^49^ HPV test quality assurance may become problematic should genuinely affordable but incompletely validated HPV test reagents, such as polymerase chain reaction primers, become available in developing countries.^7^ Incompletely validated HPV test reagents, which are nonetheless cost-additive relative to local Papanicolaou cytology, are now readily available in Vietnam^50^ and, presumably, in other developing countries.

4. *Papanicolaou cytology screening is feasible anywhere cervical screening is appropriate, and cytology will remain an essential technological component of all effective preventive solutions to the problem of cervical cancer.* Although the reproducibility and real-world benefits of increased HPV screening-test sensitivity are uncertain, the real-world drawbacks of decreased screening-test specificity and increased screening-test costs are clear. HPV tests priced at US$1/test will be cost-additive relative to Papanicolaou cytology in Vietnam,^9^ and maythereby lower screening coverage rates essential for successful cervical cancer prevention (Table I). HPV screening tests are not appropriate in the United States for women under age 30 because of unacceptably high false-positive rates attributable to HPV prevalence rates of 15%–25% among American women under age 30.^51^ Higher HPV prevalence rates in lower-resource settings will further limit the utility of HPV screening in developing countries. For example, HPV prevalence rates are 25% among Nigerian women over age 55, are higher among Nigerian women under age 55,^52^ and exceed 70% among South African women infected with human immunodeficiency virus (HIV).^53^ Because the high false-negative rate of visual screening methods among older women renders visual screening inappropriate for postmenopausal women in any setting,^54^ cytology is the only screening test appropriate for women of all age groups.

HPV and/or visual “screen and treat” are strategies that dispense, in theory, with confirmatory testing and cytology triage by providing cryosurgery to all women with positive noncytologic screening tests. However, women with positive noncytologic screening tests will be informed that they have positive screening tests for cancer, that cryosurgery will probably make it impossible for anyone to determine whether cancer is truly present, and that, if cancer is truly present, cryosurgery will be an inadequate treatment.^7^ Because individuals with positive screening tests for cancer understandably desire to know whether or not they truly have the disease, HPV and/or visual “screen and treat” strategies would necessitate regular acts of uncontested medical malpractice if ever implemented in the United States, and for corresponding reasons are unlikely to achieve adequate coverage rates in other settings.^7^ These critical shortcomings of noncytologic “screen and treat” strategies do not apply to “screen and treat” strategies incorporating at least some cytology, for which excisional treatment methods that will provide confirmatory testing can be used.^30^, ^55^ Advocates of noncytologic “screen and treat” have acknowledged the need for “screen and treat” strategies to incorporate confirmatory testing,^56^, ^57^ and it is currently difficult to envision protocols for confirmatory testing that do not incorporate a component of cytology. Novel biomarkers, including p16^INK4a^, Ki-67, and HPV L1, will probably be cost-additive and may eventually provide no decisive analytical advantages over cytology.

It is not appropriate to screen for cancer in communities without access to curative treatment services. Because communities with access to surgery and radiation therapy will have access to cytology laboratories, cytologic screening is feasible anywhere cervical screening is appropriate.^26^ Because failures of quality management have been of critical importance in past failures of cervical screening efforts,^7^ and because quality management is much more difficult for visual screening methods than for cytology,^58^ it is difficult to justify the establishment of visual screening programs in any setting where Papanicolaou screening is feasible.

Because HPV vaccination will not reduce the importance of cervical screening,^59^ cytology, in addition to its traditional role, will also be required for screening younger women in HPV-based screening programs, for screening older women in visual-based screening programs, for adequacy assessments of HPV screening tests, and for triage of women with positive HPV and visual screening tests. Allocating limited resources to invest in the infrastructure required to establish and maintain two or three screening test systems in low-resource settings, when one screening test system is both necessary and sufficient, may reduce the rate at which screening coverage is built out to high-risk demographic groups.^60^

5. *HPV vaccines will probably require booster doses, yet nonetheless may eventually fail to prevent cancer and, in the worst case, may do harm.* We will not know for many years whether HPV vaccination will prevent cancer or, in the worst case, do harm,^61^ and HPV vaccination programs may eventually prove to be costly failed public health experiments in cancer control.^62^ The most optimistic scenario of HPV vaccine effectiveness includes presumptions that the primary immunization series confers lifelong, essentially perfect protection with no need for boosters, and that there is no replacement by nonvaccine oncogenic HPV types among vaccinated women. Whether such presumptions are true is exactly what is not known.^61^ However, even the most optimistic scenario of HPV vaccine effectiveness, coupled with universal HPV vaccination, will have minimal impact on cervical cancer rates for at least 30 years after vaccine introduction.^59^, ^63^ In contrast, Papanicolaou screening reduces cervical cancer rates by 60%–90% within 3 years of its introduction.^32^ It is correspondingly unlikely that HPV vaccination will reduce cervical cancer rates among women who are also screened, and legitimate concerns regarding HPV vaccination-associated risks of venous thromboembolism, Guillain-Barré syndrome, autoimmune disorders, pancreatitis, anaphylaxis, transverse myelitis, motor neuron disease, and death must be carefully weighed against the uncertain long-term benefits of HPV vaccines.^64^, ^65^ Because the infrastructure to deliver vaccines to preadolescents and young adults is not yet established in developing countries such as Vietnam,^66^ the introduction of HPV vaccines to developing countries, even should HPV vaccines be given away for free, may compete with limited budgets for the build-out of screening services and thereby decelerate global reductions in cervical cancer-related mortality by unintentionally creating populations of women who will not be protected by either screening or vaccination.^7^, ^67^

It is unlikely that the most optimistic scenarios of HPV vaccine safety and effectiveness will be realized. Through October 2010,^68^ the U.S. National Vaccine Injury Compensation Program reported that two claims had been compensated for damages caused by HPV vaccines, and that eight additional death claims and 88 injury claims had been filed. To date, the efficacy of GlaxoSmithKline's (GSK) Cervarix™ HPV vaccine has been demonstrated for 6.4 years, and the efficacy of Merck's Gardasil™ HPV vaccine has been demonstrated for 5 years.^69^ However, HPV vaccines must confer over 30 years of essentially perfect efficacy to substantially reduce cervical cancer incidence rates,^70^ and leading scientists, including Nobel laureate Harald zur Hausen,^71^ predict that even the best-case scenario of HPV vaccination will require booster doses. If vaccine-induced protection lasts for less than 15–20 years, HPV vaccines will fail to prevent cervical cancer^69^ and, by shifting susceptibility for HPV infection to older females, may actually cause perverse effects on health outcomes.^70^ In matters pertaining to life and death, it is essential to choose the sure thing, and, by definition, dangerous to choose otherwise. With regard to cervical cancer prevention, Papanicolaou screening, done correctly, is a sure thing, but HPV vaccination, done correctly, is not.^67^ Developing countries should therefore allocate their limited resources to cervical screening, rather than HPV vaccination,^72^ until full coverage of target demographic groups by screening services has been achieved and/or the possibility has been excluded that HPV vaccination may be ineffective for cervical cancer prevention.^67^

### Step D: Activities to Encourage Others to Practice Successful “Outlier” Behaviors and Strategies of PD Group

The PD group requested meetings with the first and second BMGF Global Health Directors in 1999 and 2002, respectively, to discuss the importance of Papanicolaou screening for developing countries. Both requests were declined. Between 2001 and 2009, peer-reviewed publications documented persistent recommendations by the PD group for Papanicolaou screening in developing countries, and resistance to such recommendations by the referent group.^7^, ^9^, ^22^, ^26^, ^29^, ^30^, ^57^, ^72^–^77^ In 2006, the PD group requested support from the BMGF for a quality management initiative that aimed to improve health outcomes by using process measurements from Papanicolaou screening in Vietnam, in conjunction with root cause analyses, to assure adequate coverage of target demographic groups, valid screening test results, and appropriate followup care.^78^ In response, the BMGF informed the PD group that the BMGF would consider supporting quality management initiatives for HPV screening efforts, but not for Papanicolaou screening efforts. In June 2009, the third BMGF Global Health Director stated that the BMGF welcomed diverse viewpoints about its global health strategy and sought candid feedback.^79^ In reply, the PD group in November 2009 emailed an eight-item questionnaire, (Table II) based on findings from our PD inquiry and prior root-cause analysis,(Table I) to the BMGF global health leadership team,^80^ who declined to respond. Although lack of feedback is one of the most critical flaws in existing global aid, feedback works only if somebody listens.^6^

**Table II:** Questionnaire Submitted to the Global Health Leadership Team of the Bill & Melinda Gates Foundation^80^ in November 2009 in Response to the Foundation's June 2009 Invitation for Candid Feedback^79^

Questions to Bill & Melinda Gates Foundation global health leadership team
1. Will the Foundation consider the possibility that cytology will remain an indispensable technological component of all possible solutions to the problem of cervical cancer in developing countries?
2. Is the Foundation concerned that making big bets on unproven assumptions may introduce bias into medical research?
3. Does the Foundation share concerns, voiced by others,^43^ that methodological bias may have undermined the scientific validity of the conclusion, from the Alliance study in India, that Papanicolaou screening is ineffective for reducing mortality related to cervical cancer?^44^
4. Does the Foundation share concerns, voiced by others,^43^, ^45^ that methodological bias may have undermined the scientific validity of the highly publicized conclusion, from the Alliance study in India, that increased reductions in mortality associated with HPV screening reflect the higher sensitivity of the HPV test?^44^
5. Will the Foundation make public the scientific justification for your support of the controversial unscreened “control” group of women participating in the Alliance study in India?
6. Do educational efforts supported by the Foundation in Vietnam and other developing countries include warnings, voiced by others, that HPV vaccination will probably require booster doses,^71^ yet may still eventually fail to prevent cancer?^61^
7. Is the Foundation concerned that your high-profile support for HPV vaccination in Vietnam and other developing countries may undermine or divert funding from cervical screening programs in these settings?
8. Is the primary interest and passion of the Gates family to improve health outcomes as rapidly as possible among as many people as possible?

## Discussion

This study is limited by the axiom that not all associations are causations. The association between the Vietnam War and cervical cancer^5^ may have no causative meaning. The association between de novo establishment of Papanicolaou screening services in southern Vietnam^2^, ^9^ and subsequent reductions in cervical cancer incidence may also have no causative meaning. These limitations do not undermine the most important lesson learned from Vietnam: if our ideological commitment is to improve health outcomes as rapidly as possible among as many people as possible, then Papanicolaou screening services (with or without HPV or visual screening services) must be implemented without further delay in any setting where cervical screening is appropriate but unavailable,^26^ with consideration given to HPV vaccination after, rather than before, full coverage of target demographic groups by screening services has been achieved and/or the possibility has been excluded that HPV vaccination may be ineffective for cervical cancer prevention.^67^ Although many low-resource settings currently lack sufficient numbers of screening test collectors, cytotechnologists, and treatment facilities to provide full coverage of target demographic groups, it is paradoxical to cite shortages of required infrastructure as reasons not to develop more.^26^ Competing ideological commitments, including those embraced by grant donors, corporate sponsors, and academic investigators, (Table I) engender imprudent yet commercially useful alternative strategies prone to decelerate global reductions in cervical cancer-related mortality by suppressing the more-rapid uptake of less-expensive, home-grown, open-source cervical cancer preventive technology in favor of the less-rapid uptake of more-expensive, imported, proprietary technologies with uncertain real-world advantages and unfavorable real-world operational limitations.

Behaviors and strategies of the PD group in this study resemble those of “Searchers” described in aid-effectiveness literature, who find out what the reality is at the bottom, believe that only insiders have enough knowledge to find solutions, hope to find solutions by trial-and-error experimentation, and believe that most solutions must be home-grown.^6^ Behaviors and strategies of the referent group in this study resemble those of “Planners” described in aid-effectiveness literature, who lack knowledge of the bottom, think they already know the solutions, believe outsiders know enough to impose solutions, announce good intentions but are inattentive to implementation strategies, and raise expectations but take no responsibility for meeting them.^6^ The first guiding principle of the BMGF is that it is “driven by the interests and passions of the Gates Family,”^81^ prompting the editorial board of the British journal *Lancet* to ask, in their appeal for more transparency and accountability in BMGF decision-making, “For such a large and influential investor in global health, is such a whimsical governance principle good enough?”^82^ The BMGF's guiding principles also state that “we take risks, make big bets, and move with urgency.”^81^ In 1999, the BMGF made one of its first such bets when it allocated US$50 million to establish the Alliance on the extraordinary assumption, uninformed by root cause analysis or academic debate, that noncytologic preventive methods, rather than Papanicolaou screening, constitute the most likely solutions to the problem of cervical cancer in developing countries.^7^ It is unlikely that health outcomes among women in Vietnam would have improved to the extent they did from 1998 through 2003 if Vietnamese health leaders had followed BMGF guidance. The implications for other developing countries are unsettling.

The chief of the Global Malaria Programme for the World Health Organization (WHO), Arata Kochi, warned in 2008 that the dominance of malaria research by the BMGF risked stifling a diversity of views among scientists, with potentially far-reaching unintended consequences.^83^ Dr. Kochi warned that many of the world's leading malaria scientists are now “locked up in a ‘cartel’ with their own research funding being linked to those of others within the group,” and that “each has a vested interest to safeguard the work of the others.” He further warned that the BMGF's determination to have its favored research used to guide health recommendations “could have implicitly dangerous consequences on the policy-making process in world health.” Similar warnings appear appropriate for global cervical cancer prevention efforts. Alliance leaders have provided no answers to the question of what can be learned from the controversial no-screening arm of the Alliance India study that cannot be learned without it.^84^ Alliance cost-effectiveness studies have persistently been biased in favor of non-cytologic preventive methods,^7^, ^26^, ^74^, ^75^ and Alliance studies of HPV screening have persistently compared the performance of Papanicolaou tests analyzed in developing-country laboratories to HC2® tests shipped to American or European reference laboratories for analysis,^26^, ^36^, ^38^ suggesting that an unintended consequence of the BMGF's principles of making “big bets” “driven by the interests and passions of the Gates family” may be to introduce bias into medical research. The widely-disputed^43^, ^45^–^47^, ^85^, ^86^ 2009 conclusion from the controversial Alliance India study, which attributed improved health outcomes to nonexistent advantages in HC2® test sensitivity,^44^ was nonetheless consistent with the extraordinary Alliance founding assumption and served as the basis for policy recommendations from the U.S. National Cancer Institute that “international experts in cervical cancer prevention should now adapt HPV testing for widespread implementation…low-resource countries do not need to establish large cytologic-testing (Papanicolaou) programs whose effectiveness requires repeated screening.”^87^ These policy recommendations overlook observations that Papanicolaou cytology performed with both equal sensitivity and higher specificity than HC2® in the Alliance India study, that the price of HC2® precludes its widespread use in low-resource settings, and that readily affordable but incompletely validated HPV test reagents, such as those now readily available in Vietnam,^50^ may create “disastrous” quality management scenarios.^49^ The recommendations provide no strategy for achieving successful cervical cancer prevention using HPV screening in settings such as Nigeria (Africa's most populous country, population ∼150 million), with prohibitively high prevalence rates of HPV infection. Alliance leaders, who remain “loath” to recommend the introduction of Papanicolaou screening to high-risk communities with no cervical screening currently in place,^7^, ^73^ boasted that the Alliance India study drove “another nail in the coffin for Pap smears.”^43^ Such broadcast messages, however pernicious, nonetheless provide rationalizations for the opportunity costs associated with prioritizing research on novel preventive interventions in settings where Papanicolaou screening is feasible but unavailable. In the United States, “bad press” regarding Papanicolaou cytology^88^ was associated with subsequent increases in cervical cancer incidence and mortality among American women, raising concerns that negative media coverage may have weakened consumer demand for Papanicolaou tests, thereby lowering coverage rates.^89^ Alliance broadcasts may contribute to similar setbacks in developing countries such as Vietnam. Additional concerns derive from potential conflicts of interest associated with the partnership between the Program for Appropriate Technology in Health (PATH, Seattle WA), which others have characterized as a BMGF agent rather than an independent grantee,^25^ and the manufacturer of the HC2® and CareHPV™ tests.^7^, ^73^, ^74^, ^84^ Others have questioned whether major public health organizations are able to effectively manage conflicts of interest.^90^

The methods by which HPV vaccines have been marketed in the United States have generated controversy^91^ and presented important challenges to medical professionalism.^92^ In 2006, before the first Phase III trials of HPV vaccination with clinically relevant endpoints had been reported, the U.S. Food and Drug Administration licensed Merck's Gardasil™ HPV vaccine for use in the United States, and the BMGF allocated US$27.8 million to promote HPV vaccination in Vietnam,^93^ India, Uganda, and Peru. In 2006, the BMGF endowment held between US$100 million and US$1 billion of Merck stock,^94^ a position it sold in 2009.^95^ The current BMGF Global Health Director, a former GSK executive, holds significant equity positions in GSK.^96^ The Office of Technology Transfer of the U.S. National Institutes of Health (NIH) reports that, based on royalties from product sales, Gardasil™ and GSK's Cervarix™ HPV vaccines have become the NIH's #1 revenue generators.^97^ Neither BMGF-funded advocacy literature promoting HPV vaccination and visual screening in Vietnam^98^ nor the NIH FactSheet about HPV vaccination^99^ includes the important educational messages that HPV vaccines will probably require booster doses^71^ yet may still eventually fail to prevent cancer and, in the worst case, may do harm.^61^ In 2008, Vietnam's Ministry of Health approved Cervarix™ for females ages 9 through 55 and Gardasil™ for females ages 9 through 26, leading to allegations that GSK and Merck had used inappropriate lobbying campaigns to procure Vietnamese Ministry approval.^100^ The WHO position paper on HPV vaccination provides ambiguous guidance, stating that “HPV vaccines should be introduced as part of a coordinated strategy to prevent cervical cancer and other HPV-related diseases…. Also, the introduction of HPV vaccine should not undermine or divert funding from effective screening programmes for cervical cancer.”^101^ The health insurer association of Switzerland agreed to finance HPV vaccination of Swiss citizens, but in exchange asked the federal benefit commission to increase the time between two reimbursed Papanicolaou tests.^102^ It is correspondingly difficult to presume that the introduction of HPV vaccines to lower-resource settings will not divert funding and/or consumer demand away from cervical screening services.

The poor suffer disproportionately not only because of the world's indifference to their poverty, but also because of ineffective efforts by those who do care.^6^ Remarkably, the BMGF promotes HPV vaccination, but not Papanicolaou screening, in developing countries such as Vietnam. Risks associated with bets placed on HPV vaccination appear to be socialized, and are particularly unsettling in light of the observed deceleration in reductions of cervical cancer incidence in Vietnam. In 2010, the government of India, responding to concerns regarding HPV vaccine safety and efficacy presented in a memorandum from 68 Indian human rights groups, women's groups, academics, and individuals, suspended BMGF-supported demonstration projects of HPV vaccination in India.^103^ Lessons learned from Vietnam reinforce appeals voiced by the *Lancet* editorial board that “now is an inflection point in the BMGF's history, a moment when change is necessary.”^82^ Global health leaders should embrace an ideological commitment to the appropriate public health goal of improving health outcomes as rapidly as possible among as many people as possible, and assimilate the policy implications of that commitment. If competing ideological commitments, including those embraced by the BMGF,^27^ can be set aside, future global cervical cancer prevention efforts can become more effective than those of the past.
